# Association of childhood BMI trajectory with post-adolescent and adult lung function is mediated by pre-adolescent DNA methylation

**DOI:** 10.1186/s12931-022-02089-4

**Published:** 2022-07-29

**Authors:** Rutu Rathod, Hongmei Zhang, Wilfried Karmaus, Susan Ewart, Fawaz Mzayek, S. Hasan Arshad, John W. Holloway

**Affiliations:** 1 Division of Epidemiology, Biostatistics and Environmental Health, School of Public Health, University of Memphis, Memphis, TN 38152-0001 USA; 2 College of Veterinary Medicine, Michigan State University, East Lansing, MI USA; 3 NIHR Southampton Biomedical Research Centre, University Hospital Southampton, Southampton, UK; 4 Clinical and Experimental Sciences, Faculty of Medicine, University of Southampton, Southampton, UK; 5 David Hide Asthma and Allergy Research Centre, Isle of Wight, UK; 6 Human Development and Health, Faculty of Medicine, University of Southampton, Southampton, UK

**Keywords:** BMI trajectory, DNA methylation, IOWBC, Lung function

## Abstract

**Background:**

Body mass index (BMI) has been shown to be associated with lung function. Recent findings showed that DNA methylation (DNAm) variation is likely to be a consequence of changes in BMI. However, whether DNAm mediates the association of BMI with lung function is unknown. We examined the mediating role of DNAm on the association of pre-adolescent BMI trajectories with post-adolescent and adulthood lung function (forced expiratory volume (FEV_1_), forced vital capacity (FVC), and FEV_1_/FVC).

**Methods:**

Analyses were undertaken in the Isle of Wight birth cohort (IOWBC). Group-based trajectory modelling was applied to infer latent BMI trajectories from age 1 to 10 years. An R package, *ttscreening*, was applied to identify CpGs at 10 years potentially associated with BMI trajectories for each sex. Linear regressions were implemented to further screen CpGs for their association with lung function at 18 years. Path analysis, stratified by sex, was applied to each screened CpG to assess its role of mediation. Internal validation was applied to further examine the mediation consistency of the detected CpGs based on lung function at 26 years. Mendelian randomization (MR-base) was used to test possible causal effects of the identified CpGs.

**Results:**

Two BMI trajectories (high vs. low) were identified. Of the 442,475 CpG sites, 18 CpGs in males and 33 in females passed screening. Eight CpGs in males and 16 CpGs in females (none overlapping) were identified as mediators. For subjects with high BMI trajectory, high DNAm at all CpGs in males were associated with decreased lung function, while 8 CpGs in females were associated with increased lung function at 18 years. At 26 years, 6 CpGs in males and 14 CpGs in females showed the same direction of indirect effects as those at 18 years. DNAm at CpGs cg19088553 (*GRIK2*) and cg00612625 (*HPSE2)* showed a potential causal effect on FEV_1_.

**Conclusions:**

The effects of BMI trajectory in early childhood on post-adolescence lung function were likely to be mediated by pre-adolescence DNAm in both males and females, but such mediation effects were likely to diminish over time.

**Supplementary Information:**

The online version contains supplementary material available at 10.1186/s12931-022-02089-4.

## Introduction

Obesity has reached epidemic proportions and its increasing prevalence has emerged as an important public health problem [1, 2] despite increasing awareness and the advent of many behavioral interventions. Its pathophysiology involves complex interactions between genetic, environmental, and biological factors [2]. Obesity increases the risk of many chronic diseases among children and adults [3].

Asthma and chronic obstructive pulmonary disease (COPD) are common chronic respiratory conditions. Lung function tests are used as a diagnostic measure for these conditions, and these tools along with asthma and COPD are influenced by genetic and environmental factors [4–6]. Obesity is known to be associated with decline of lung function parameters [7–9], thereby contributing to the burden of respiratory conditions [10]. Sex differences in lung function are observed in the post-pubertal period and continue in adulthood [11].

Several recent studies have suggested a role for epigenetic programming in obesity [12–14] and lung development [15]. One of the most widely studied epigenetic mechanisms is DNA methylation (DNAm), which is influenced by both genetic and environmental exposures [16, 17] and regulates gene expression. DNAm at specific cytosine-phosphate-guanine (CpG) sites has been found to be associated with BMI [18–25] and lung function [26–29]. Recent investigations have suggested that changes in DNAm in blood and in adipose tissue associated with BMI are primarily the consequence of, as opposed to causal for BMI [30, 31].

Given that a decline in lung function has been demonstrated in subjects with high BMI, and DNAm is associated with both BMI and lung function, we hypothesized that DNAm mediates the association of BMI trajectories before adolescence with post-adolescent and adulthood lung function. Path analyses were utilized to examine the mediation effects of DNAm. Since sex differences in lung function exist, all analyses were stratified by sex.

## Methods

### Study population

From January 1, 1989 to February 28, 1990, a whole population birth cohort was established on the Isle of Wight, UK, to prospectively study the natural history of allergic diseases among children. After exclusion of adoptions, perinatal deaths, and refusal to follow up, 1,456 newborns were enrolled in the study. These newborns were followed at ages 1, 2, 4, 10, 18 and 26 years. Detailed interviews and examinations were completed for each participant child at each follow-up. The IoW birth cohort (IOWBC) has been described in detail elsewhere [32, 33]. All the subjects included in this study are from the F1 generation with no siblings.

### Data collection: outcome, exposures, covariates

Lung function parameters were measured using a Koko Spirometer and software with a portable desktop device (both PDS Instrumentation, Louisville, KY, USA), according to American Thoracic Society guidelines [34]. Lung function measurements performed at 18 years of age were included in this study, including forced expiratory volume in 1 s (FEV_1_), forced vital capacity (FVC), and FEV_1_/FVC ratio.

The height and weight of each participant was assessed at ages 1, 2, 4, 10, 18 and 26 years. Body mass index (BMI) was calculated using weight in kilograms divided by height in meters-squared at each age. Information regarding sex was assessed based on hospital records at birth. Information regarding age at pubertal events, i.e., age at onset of voice deepening in males and age at onset of menarche in females, was obtained from questionnaire data. Active smoking status at 18 years was recorded as either never smoker, current smoker, or past smoker. Information obtained for tobacco smoke exposure from mother, father, or others at ages 1, 2, and 4 years was used to determine second-hand smoke exposure until 10 years of life.

Socioeconomic status was defined using a composite variable, ‘‘Family social status cluster’’ as previously described [35]. Briefly, family social status was clustered using three variables: (a) British socioeconomic classes derived from parental occupation reported at birth; (b) the number of children in the index child’s bedroom (collected at age 4 years); and (c) family income at age 10 years. This yielded six groups, one clearly ‘‘highest’’ and one clearly ‘‘lowest’’ status, and four ‘‘middle’’ clusters representing a diversity of middle-class living conditions, these were then simplified to three groups by combining the middle four groups as “middle” economic status groups. This composite variable captures the family social class across the entire study period.

### DNA methylation

As described previously [36–39] DNA was extracted from whole blood at 10 and 26 years and genome-wide DNAm for each CpG was assessed using either Illumina Infinium HumanMethylation450 or the Methylation EPIC BeadChips (Illumina, Inc, San Diego, CA, USA), which interrogate > 484,000 and > 850,000 CpG sites, respectively with multiple identical control samples assigned to each bisulfite conversion batch for assessment of assay variability. DNAm data were preprocessed using the CPACOR pipeline for data from both platforms (HumanMethylation450 and MethylationEPIC) [40]. Specifically, an R package, *minfi* was used to quantile-normalize the DNAm intensity data [40, 41]. Quantile-normalized intensities were then used to calculate beta (*β*) values, which represent proportions of intensity of methylated (*M*) over the sum of methylated and unmethylated (*U*) sites/probes (*β* = *M*/ [c + *M* + *U*], where c is a constant to prevent zero in the denominator if *M* + *U* is too small). Beta values close to 0 or 1 tend to suffer from severe heteroscedasticity, and base-2 logit transformed beta values (denoted as M-values) have been demonstrated to perform better in differential analysis of methylation levels [42]. Therefore, methylation levels in the analysis are represented using M-values.

Probes not reaching a detection p-value of 10^–16^ in at least 95% of samples were excluded. Samples with a low quality of DNAm measurement were excluded based on application of a comparable criterion. CpGs on the sex chromosomes were excluded from our analyses to avoid bias. Probes that contained single nucleotide polymorphisms (SNPs) within 10 base pairs of a targeted CpG site with a minor allele in at least 0.7% subjects (corresponding to at least 10 subjects in IOWBC with n = 1,456) were excluded due to their influence on DNAm. CpGs that passed quality control and were common between the two platforms (450 K and EPIC) were included in the analyses.

To describe chip‐to‐chip and other technical variations, latent variables represented by principal components (PCs) were used. PCs were inferred based on control probes. Since DNAm data were from two different platforms, PCs were determined based on DNAm at shared control probes. In total, 195 control probes were shared between the two platforms (450 K and EPIC) and used to calculate the control probe PCs and the top 15 were used to represent latent batch factors [40].

Since blood is a mixture of functionally and developmentally distinct cell populations [43], adjusting for cell type compositions in DNAm measured from blood samples potentially removes confounding of cell heterogeneity [44]. To this end, we estimated cell type proportions using the method proposed by Jaffe and Irizarry [45], adapted from Houseman et al. [46], using the Bioconductor *minfi* package [41]. The estimated cell type proportions of CD4 + T cells, natural killer cells, neutrophil, B cells, monocytes, and eosinophil cells were included as confounding factors in the analyses.

### Genome-wide RNA-seq gene expression data generation

Gene expression levels were measured in peripheral blood samples collected at 26 years from the IOWBC using paired-end (2 × 75 bp) RNA sequencing with the Illumina Tru-Seq Stranded mRNA Library Preparation Kit with IDT for Illumina Unique Dual Index (UDI) barcode primers, following the manufacturer’s recommendations. All samples were sequenced twice using the identical protocol and for each sample the output from both runs were combined. FASTQC were run to assess the quality of the FASTQ files (https://www.bioinformatics.babraham.ac.uk/projects/fastqc/). Reads were mapped against Human Genome (GRch37 version 75) using HISAT2 (v2.1.0) aligner [47]. The alignment files, produced in the Sequence Alignment Map (SAM) format, were converted into the Binary Alignment Map (BAM) format using SAMtools (v1.3.1) [48]. HTseq (v0.11.1) was used to count the number of reads mapped to each gene in the same reference genome used for alignment [49]. Normalized read count FPKM (Fragments Per Kilobase of transcript per Million mapped reads) were calculated using the countToFPKM package (https://github.com/AAlhendi1707/countToFPKM) and their log-transformed values were used for data analysis.

### Statistical analysis

Chi-square tests for categorical variables and one-sample t-tests for continuous variables were applied to assess if the study sample reasonably represents the complete cohort. All the analyses were stratified by sex due to sex differences in pulmonary function tests.

#### BMI trajectories

BMI values at ages 1, 2, 4 and 10 years were used to determine BMI trajectories separately for each sex. BMI developmental paths in the form of trajectories across childhood were identified by applying a group-based trajectory modelling, also referred to as a semiparametric mixture model [50, 51], using PROC TRAJ in SAS [52]. The group-based trajectory method presumes that the data comprises of latent distinct groups (trajectories) that best summarize the distinct features as parsimonious as possible [50, 51]. Models with one to three groups were estimated for linear and quadratic terms. The smallest Bayesian Information Criterion value was used to select the best fit model. Individuals were assigned to one of the trajectories/groups based on their highest estimated group-membership probabilities.

#### Association of BMI trajectories with lung function parameters

We used multivariable linear regression to evaluate the association of BMI-trajectory (independent variable) with markers of lung function at 18 years (dependent variable) along with covariates and confounders potentially associated with lung function in the model: SES, active smoking status at 18 years, height at 10 years, maternal smoking during pregnancy, duration of breastfeeding (in weeks), parental history of asthma and age at pubertal events. For males, age at voice deepening, and for females, age at menarche were included in the model.

#### Screening for CpGs related to BMI trajectories

Regression of M-values of DNAm at each CpG site on the aforementioned 15 PCs and the 6 cell type proportions [46] was done to obtain batch- and cell-type-adjusted DNAm (residuals) for each sex, which were used in subsequent analyses. An R package, *ttScreening* [53], was applied to screen CpGs at 10 years that were potentially associated with BMI trajectory groups, separately for each sex. The method implemented in this package has been detailed elsewhere [53]. Briefly, it is built upon robust linear regressions utilizing training and testing samples via the concept of cross-validation to screen CpGs. The split between training and testing samples was random such that 2/3^rd^ of the data were randomly selected as the training set and the remaining were used as testing data, following the suggestion in the literature to optimize power [54]. In addition, with the inclusion of surrogate variables, the method can account for variation in methylation introduced by other unknown factors. The minimum frequency of selecting an informative CpG site was set at 50%, i.e., a CpG site gained statistical significance in at least 50% of the randomly selected training and testing data set pairs [53]. CpGs that passed the screening were treated as BMI-trajectory-associated-CpGs. This final pool of CpG sites is the result of the *ttScreening* approach, which was further analyzed with the full dataset for regression coefficients and p-values.

#### Screening for BMI-trajectory-associated CpGs with lung function parameters

We used multivariable linear regression to evaluate the association of BMI-trajectory-associated-CpGs (independent variable) with lung function parameters at 18 years (dependent variables). Each of the parameters, i.e., FEV_1_, FVC and FEV_1_/FVC ratio, were tested in separate models. The covariates and confounders potentially related to lung function were included in the model: BMI trajectory groups, SES, active smoking status at 18 years, height in centimeters at 18 years and age at pubertal events. Age at voice deepening for males, and age at menarche for females were used for pubertal events in the model. Statistical significance for this in-depth screening process was set at 0.05. CpGs that showing statistical significance in this multivariable regression analysis were further included in path analyses to estimate magnitude and statistical significance of mediation effects.

#### Path analyses

**Fig. 1:**
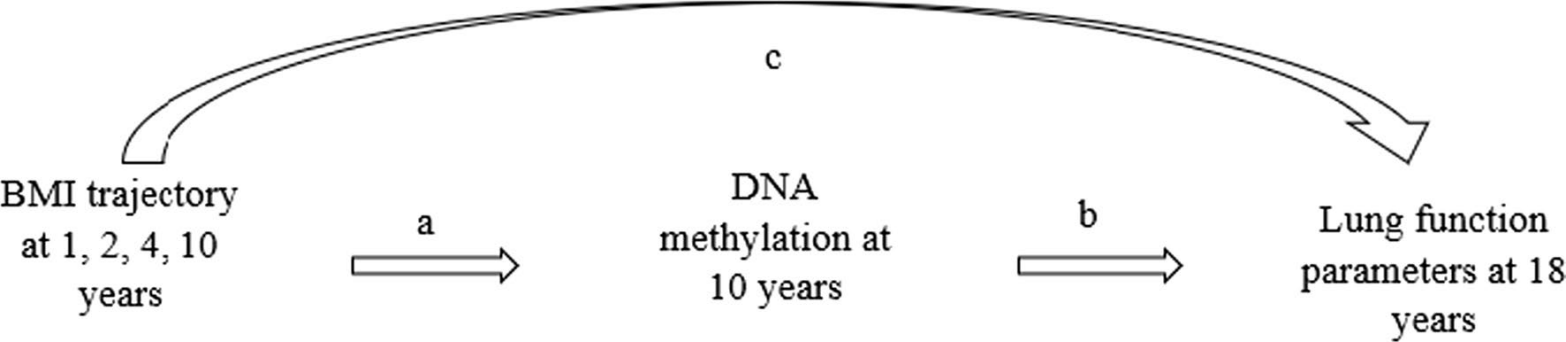
Path analysis model showing DNAm at 10 years as the mediator between the association of BMI trajectory at earlier childhood and lung function parameters (FEV_1_, FVC, FEV_1_/FVC ratio) at post-adolescence. **a** Effects of BMI-trajectories on methylation of CpGs, controlled for secondhand smoking status before 10 years. **b** Effects of DNAm on the lung function, controlled for BMI trajectories, socio-economic status (SES), active smoking status at 18, height at 18 years, pubertal events (age at onset of voice deepening in males and age at onset of menarche in females). **c** Direct effects of BMI trajectories on lung function parameters

Path analyses were used to assess the magnitude and statistical significance of mediation effects of DNAm at 10 years in the association between BMI trajectories from ages 1 to 10 years based on BMI at1, 2, 4, and 10 years and lung function parameters at 18 years (Fig. 1). Potential confounders were included in each path. Goodness of fit criteria was evaluated using Chi-square test p-value > 0.05, RMSEA < 0.05, CFI > 0.95 [55]. The direct and indirect estimates, i.e., the path coefficients, represent the partial correlation between the independent and dependent variables after adjusting for confounders and covariates [39]. An R package, *MplusAutomation*, was applied to iteratively call *MPlus* from R to perform path analyses with each of the CpGs as a mediator (Fig. 1) [56].

#### Further assessment of findings at a later age

For the identified CpGs showing mediation effects, we examined whether the effects were consistent for lung function at a later age. To this end, CpGs identified as mediators for lung function parameters at 18 years were further evaluated for their mediation effects for lung function at age 26 years. The same set of covariates were used in the path analysis models except for the variable active smoking; for this replication assessment, to be consistent, active smoking status was recorded at age 26 years.

#### Mendelian randomization

The web tool *MR-base* (https://www.mrbase.org/) [57, 58], was used to infer causal relationship between the identified mediating CpGs (obtained from path analyses) (exposure) and lung function parameters (outcome).

#### Association between DNAm and gene expression

Genes annotated to the identified CpGs showing mediation effects were extracted along with information such as gene location, and chromosome number from the Illumina's manifest file or UCSC genome browser (https://genome.ucsc.edu/). To assess the biological relevance of CpGs showing mediating effects, linear regressions were applied to test the association of DNAm (in M- values; independent variable) at 26 years at each CpG site with expression of its neighboring genes (250 k base pairs [bps] upstream and 250 k bps downstream of the CpG site) in blood at the same age. A window size of ± 250 k bps for expression quantitative methylation analysis was used following the suggestion of Reese et.al. [59]. Paired DNAm and expression data of n = 140 subjects were included in the analyses. As we previously have found the association between gene expression and DNAm to be different in both males and females, the analysis was stratified by sex [36, 38].

## Results

Participants in IOWBC with DNAm data available, i.e., 190 males and 140 females were included our study. The analytical subsamples represented the complete IOWBC (n = 1456) with respect to lung function parameters, demographic variables, and other covariates included in our study (p > 0.05) (Table 1).

**Table 1:** Comparison of analytical subsample with complete cohort

Variables		Males			Females		
Categorical variables		Subsample (n = 190); n (%)	Complete cohort (n = 786); n (%)	p-value	Subsample (n = 140); n (%)	Complete cohort (n = 750); n (%)	p-value
Socio-economic status	Low	23 (12.23)	106 (15.5)	0.46	22 (15.83)	103 (15.30)	0.72
	Mid	150 (79.79)	517 (75.58)		104 (74.82)	520 (77.27)	
	High	15 (7.98)	61 (8.92)		13 (9.35)	50 (7.43)	
Active smoking status (18 years)	Past	96 (51.34)	340 (54.23)	0.43	77 (55.4)	323 (50.16)	0.53
	Current	50 (26.74)	176 (28.07)		37 (26.62)	192 (29.81)	
	Never	41 (21.93)	111 (17.7)		25 (17.99)	129 (20.03)	
Active smoking status (26 years)	Yes	58 (37.91)	146 (31.06%)	0.12	40 (32)	176 (31.37)	0.89
	No	95 (62.09)	324 (68.94%)		85 (68)	385 (68.63)	
Second-hand smoking (10 years)	Yes	106 (56.68)	489 (64.26%)	0.05	80 (57.55)	445 (61.38)	0.40
	No	81 (43.32)	272 (35.74%)		59 (42.45)	280 (38.62)	

Continuous variables	Mean ± SD	Mean ± SD		Mean ± SD	Mean ± SD	p-value
Age of puberty	14.1 ± 1.21	14.24 ± 1.24	0.11	12.6 ± 1.36	12.72 ± 1.42	0.3
Height at 18 years	177.40 ± 6.82	178.24 ± 6.87	0.09	164.14 ± 6.45	164.73 ± 6.33	0.28
FEV_1_ at 18 years	4.65 ± 0.62	4.6 1 ± 0.67	0.38	3.5 ± 0.41	3.47 ± 0.54	0.39
FVC at 18 years	5.37 ± 0.73	5.36 ± 0.78	0.89	3.98 ± 0.45	3.95 ± 0.58	0.41
FEV_1_/FVC (18 years)	0.87 ± 0.08	0.87 ± 0.11	0.89	0.88 ± 0.06	0.89 ± 0.12	0.08

**Fig. 2:**
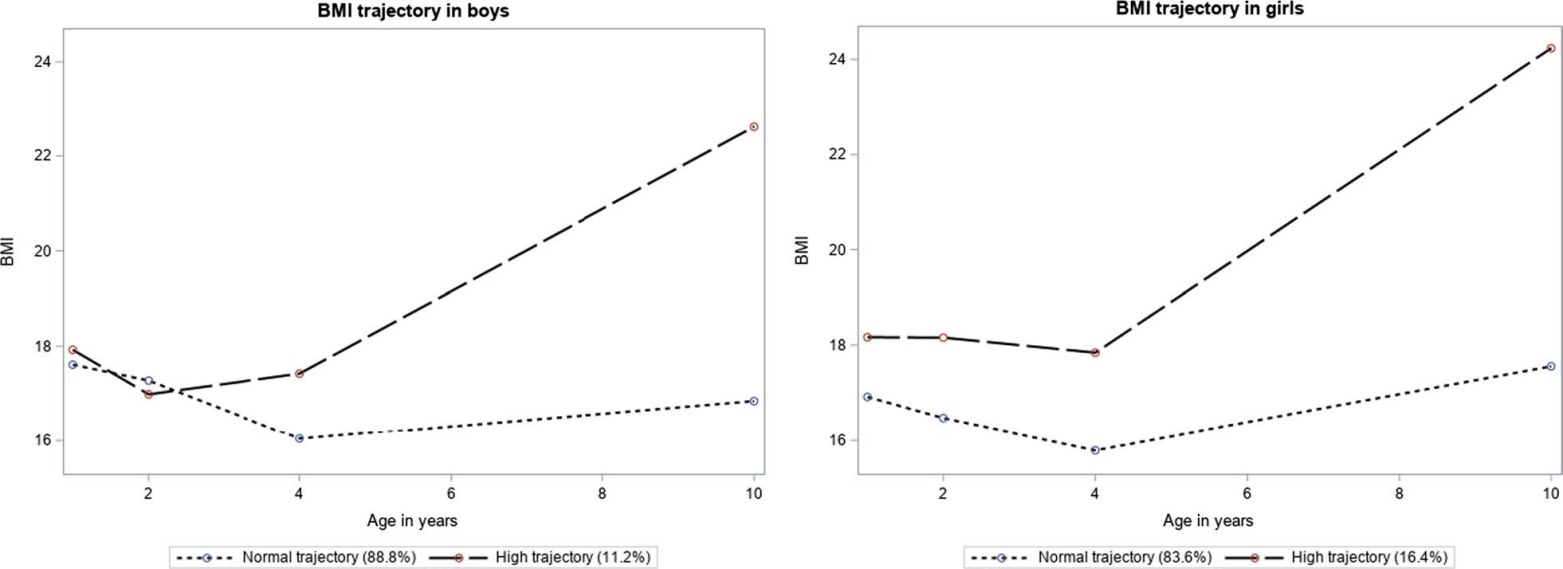
BMI trajectories across first 10 years of life in boys and girls respectively

Two BMI trajectories were identified for both sexes that best summarized the complex developmental course of BMI across the first 10 years of life based on patterns observed in IOWBC. The trajectories were labeled as ‘normal’ (dotted lines) and ‘high’ (dashed lines) (Fig. 2).

BMI trajectories were associated with at least one lung function parameter, FVC, FEV_1_, or FEV_1_/FVC, at different ages (18 and 26 years) (Additional file 1: Table S1). For males, BMI trajectories were significantly associated with FEV_1_ and FVC at one or both ages, while for females, the association was statistically significant for FEV_1_/FVC at age 26 years. At 18 and 26 years FEV_1_ and FVC in males with high BMI trajectory were lower on average than those in normal BMI trajectory after controlling for confounders and covariates, and for FEV_1_/FVC, the same pattern was observed in females (Additional file 1: Table S1).

After preprocessing, 442,475 CpGs were included in subsequent analyses. To identify candidate CpGs potentially associated with BMI trajectory groups, *ttScreening* was applied stratified for sex using residuals (batch- and cell-type-adjusted DNAm) at 10 years of age. In total, 159 CpGs in males and 212 CpGs in females passed screening. These CpGs were treated as potentially BMI trajectory associated CpGs and were included in subsequent analyses.

For each CpG that passed screening, its association with lung function parameters at age 18 years was further evaluated in separate models. This is a subsequent step of screening of CpGs for the purpose of fitting a valid path analysis model. After controlling for potential confounders, at significance level of 0.05, 18 CpGs in males (union of nine for FEV_1_, four for FVC, and six for FEV_1_/FVC) and 33 CpGs in females (union of 17 for FEV_1_, eight for FVC, and 18 CpGs for FEV_1_/FVC) were found to be associated with lung function parameters at 18 years.

These 18 CpGs in males and 33 CpGs in females were then tested for their mediation effects on the association of BMI trajectories at ages 1, 2, 4, 10 years with each of the lung function parameters at 18 years. In total, 24 CpGs were identified. In particular, eight CpGs in males (union of four for FEV_1_, two for FVC, and three for FEV_1_/FVC) and 16 CpGs in females (union of six for FEV_1_, four for FVC, and nine for FEV_1_/FVC) showed mediation effects for lung function parameters at 18 years (Table 2). There were no overlapping CpGs between males and females.

**Table 2:** Indirect and direct effects of childhood BMI trajectories on lung function parameters at post-adolescent and adulthood via pre-adolescent DNAm along with the information on locations of the identified CpG sites

Outcome	Sex	CpG sites	Effects at 18 years*				Effects at 26 years**				Genes	Gene Location	Chr. ^#^
			Indirect eff. ^$^	p-value	Direct eff.^¥^	p-value	Indirect eff. ^$^	p-value^@^	Direct eff.^¥^	p-value			
FEV_1_	Males	**cg00612625**	− **0.16**	0.02	− 0.14	0.55	**− 0.001**	0.99	− 0.57	0.03	*HPSE2*	Body	10
		**cg21883115**	**− 0.14**	0.03	− 0.16	0.50	**− 0.04**	0.48	− 0.53	0.03	*MAGI3*	–	1
		cg00509207	− 0.14	0.04	− 0.14	0.52	0.02	0.83	− 0.60	0.01	*TSPAN11*	TSS1500	12
		**cg06710672**	**− 0.13**	0.03	− 0.14	0.54	**− 0.15**	**0.05**	− 0.41	0.05	*CACNA1H*	–	16
	Females	**cg09005221**	**− 0.17**	0.03	0.28	0.27	**− 0.13**	0.16	0.15	0.64	*DIO2*	Body	14
		**cg14654082**	**0.21**	0.04	− 0.09	0.74	**0.23**	0.07	− 0.16	0.62	*CAMTA1*	Body	1
		**cg13314614**	**0.28**	0.004	− 0.19	0.45	**0.15**	0.12	− 0.14	0.66	*DHRS3*	Body	1
		**cg19088553**	**0.16**	0.03	− 0.08	0.76	**0.17**	**0.05**	− 0.19	0.53	*GRIK2*	Body	6
		**cg19411557**	**0.25**	0.04	− 0.15	0.55	**0.16**	0.17	− 0.09	0.76	*RP11− 285B24.1*	–	3
		**cg04206296**	**0.17**	0.03	− 0.08	0.76	**0.15**	**0.04**	− 0.15	0.63	*DEGS1*	TSS1500	1
FVC	Males	cg10337540	− 0.12	0.04	− 0.17	0.40	0.01	0.88	− 0.73	0	*LHFP*	Body	13
		**cg00612625**	**− 0.15**	0.02	− 0.14	0.46	**− 0.005**	0.95	− 0.71	0	*HPSE2*	Body	10
	Females	cg26606556	− 0.19	0.03	0.40	0.05	0.08	0.4	0.14	0.59	*FARSA*	TSS200	19
		**cg18860868**	**− 0.14**	0.04	0.37	0.07	**− 0.12**	0.1	0.34	0.19	*FBXO21*	Body	12
		cg00647820	− 0.16	0.03	0.41	0.04	0.06	0.47	0.16	0.54	*DHX58*	Body	17
		**cg21909286**	**0.16**	0.02	0.07	0.73	**0.04**	0.68	0.19	0.39	*BEGAIN*	–	14
FEV_1_/FVC	Males	**cg15594471**	**− 0.21**	0.02	0.25	0.25	**− 0.17**	0.14	0.30	0.30	*RP11-856F16.2*	–	11
		**cg15628222**	**− 0.16**	0.03	0.18	0.42	**− 0.15**	0.12	0.29	0.34	*TRAM1*	TSS1500	8
		**cg18078387**	**− 0.16**	0.03	0.19	0.36	**− 0.18**	0.14	0.21	0.44	*SCIN*	TSS200	7
	Females	**cg18164362**	**0.24**	0.04	− 0.47	0.14	**0.15**	0.24	− 0.61	0.13	*RP11-163,019.10*	–	11
		**cg02710571**	**0.29**	0.02	− 0.47	0.09	**0.21**	0.08	− 0.67	0.04	*CCDC140*	5’UTR	2
		**cg14880800**	**0.28**	0.02	− 0.53	0.05	**0.21**	0.08	− 0.67	0.02	*DNAH5*	TSS1500	5
		cg14654082	0.23	0.03	− 0.44	0.12	− 0.08	0.65	− 0.38	0.32	*CAMTA1*	Body	1
		**cg13314614**	**0.20**	0.04	− 0.44	0.11	**0.05**	0.67	− 0.50	0.17	*DHRS3*	Body	1
		**cg26606556**	**0.24**	0.04	− 0.46	0.10	**0.08**	0.48	− 0.53	0.18	*FARSA*	TSS200	19
		cg17023770	0.25	0.02	− 0.49	0.07	− 0.02	0.86	− 0.42	0.26	*PCDHB15*	TSS1500	5
		**cg20015855**	**0.25**	0.04	− 0.46	0.11	**0.16**	0.09	− 0.61	0.09	*CORO2B*	Body	15
		**cg11763866**	**0.24**	0.04	− 0.46	0.09	**0.29**	**0.04**	− 0.78	0.007	*PPFIA1*	–	11

*For analyses at 18 years, path analyses were adjusted for second-hand smoke exposure before 10 years, socio-economic status (SES), active smoking status at 18 years, height at 18 years, age of pubertal events (age at onset of voice deepening for males and age at onset of menarche for females)

**Analyses at 26 years used similar covariates: second-hand smoke exposure before 10 years, socio-economic status (SES), active smoking status at 26 years, height at 18 years, age of pubertal events (age at onset of voice deepening for males and age at onset of menarche for females)

Underlined CpGs are common CpGs between two lung function parameters in same sex

CpGs in bold font show the same direction of indirect effects at 26 years as those at 18 years

$Indirect eff.: Estimates of indirect effects (coefficients)

¥Direct eff.: Estimates of direct effects (coefficients)

@ In the replication analysis, p-values ≤ 0.05 are in bold font and p-values ≤ 0.1 are underlined

#Chr.: Chromosome

To help understand the mediating effects of DNAm, Additional file 1: Table S2 shows the direct effects for each path at each of the 24 CpG sites for all lung function parameters at 18 years. At all these CpGs except for cg00647820 in females for FVC, BMI trajectory showed only indirect effects (via DNAm at these CpGs) on the variation of lung function measurement at 18 years, and no statistically significant direct effects were observed. At eight CpG sites in males (union of four for FEV_1_, two for FVC and three for FEV_1_/FVC), the coefficients suggested that high BMI trajectory was associated with high DNAm, which was further linked to decreased lung function at 18 years (Additional file 1: Table S2). At 13 of the 16 CpGs in females (union of five for FEV_1_, one for FVC and nine for FEV_1_/FVC), the coefficients suggested that high BMI trajectory was associated with increased lung function at age 18 years via high DNAm at those CpGs (Additional file 1: Table S2).

To further assess the consistency of the discovered 24 CpGs (8 in males) regarding their mediation effects, we tested them using the same lung function parameters measured at 26 years (Table 2). At 26 years, 6 (75%) of the 8 discovered CpG sites in males (union of three for FEV_1_, one for FVC and three for FEV_1_/FVC) and 14 (87.5%) of the 16 discovered CpG sites in females (union of six for FEV_1_, two for FVC and seven for FEV_1_/FVC) showed the same direction of indirect effects as those discovered at age 18 years, with four CpGs (one in males) showing statistical significance at the significance level of 0.05 and five CpGs (one in males) at the level of 0.1 (Table 2).

The 20 CpGs showing consistency at both time points were further evaluated for their causal associations with lung function using Mendelian Randomization. Of these 20 CpGs, DNAm at cg19088553 (on gene *GRIK2*) and cg00612625 (*HPSE2)* demonstrated a potential causal effect on FEV_1_ (p-value = 0.02 for both CpGs) (Additional file 1: Table S4).

To assess the biological relevance of CpGs showing mediating effects for lung function parameters at 18 and 26 years, we evaluated the association of DNAm at the identified 20 CpGs (6 in males) with the expression of genes that the CpGs were mapped to as well as nearby genes (± 250 k bps). In this analysis, both DNAm and gene expression levels were assessed at age 26 years. Significant effects of DNAm were observed at 4 CpGs on their association with expression of 25 genes in males, and at 10 CpGs with expression of 78 genes in females (Table 3 and Additional file 1: Table S3). Of the 25 genes in males, increased DNAm was associated with increased expression of 21 genes, and of the 78 genes in females, increased DNAm was associated with increased expression of 33 genes. Unfortunately, for the two CpGs, cg19088553 (*GRIK2*) and cg00612625 (*HPSE2)* showing potential causal effects, we were not able to assess their biological relevance due to lack of expression data for *GRIK2* and *HPSE2* in whole blood.

**Table 3:** Associations of DNAm with expression of mapped genes within 500k bps

Sex	CpG	Gene*	DNAm effect	SE	p-value	Approximate distance between CpG site and gene (in bps)
Males	cg21883115^#^	*LARP7*	0.9	0.36	0.014	165–183k
	cg15628222	*LINC00469*	2.11	0.82	0.014	232–246k
		***TRAM1***	0.87	0.47	0.072	33
	cg15594471^#^	*CWC15*	0.52	0.13	0.0003	175–187k
	cg10337540	*KLHL11*	1.54	0.68	0.029	45–60k
	cg06710672^#^	*TRIP13*	− 0.54	0.25	0.033	215–241k
	cg00509207^#^	*DDX11*	1.45	0.70	0.044	147–178k
Females	cg00647820	*CHRNA9*	− 0.75	0.31	0.019	76–96k
	cg02710571^#^	*SUSD4*	− 0.71	0.30	0.020	229–242k
	cg04206296	*CNIH4*	0.22	0.09	0.019	172–196k
		***DEGS1***	0.17	0.09	0.071	7–97
	cg09005221^#^	*DYNLRB2*	− 0.99	0.33	0.004	92–103k
	cg13314614	*PARVA*	1.12	0.40	0.006	102–245k
		***DHRS3***	0.66	0.23	0.006	23–25
	cg14654082	*TMEM88*	0.69	0.28	0.016	37–39k
	cg14880800^#^	*COX10*	0.33	0.15	0.030	25–165k
	cg17023770^#^	*SLC25A36*	0.52	0.24	0.030	34–71k
	cg18164362^#^	*EFCAB1*	− 1.99	0.88	0.026	224–244k
	cg20015855	*AUTS2*	− 1.02	0.28	0.0004	125–242k
	cg21909286^#^	*ARHGAP42*	− 1.03	0.42	0.016	73–220k
	cg26606556	*MAST1*	− 1.07	0.40	0.008	62–96k
		***FARSA***	− 0.58	0.17	0.001	

For each CpG, the neighboring gene with the largest absolute value of regression coefficient was shown along with their mapping genes if the p-value is (marginally) significant. The full list of CpGs is in Additional file 1: Table S3

*Genes in first row are the nearby genes with largest absolute effect size and genes in second row (in bold font) for same CpG is its corresponding mapped gene

^#^Gene expression data was not available for mapping genes at these CpGs

## Discussion

We assessed the direct and indirect effects of BMI trajectory in childhood on post-adolescence lung function parameters via DNAm. DNAm at eight CpGs in males and 16 CpGs in females showed mediation effects in the association of BMI trajectory and lung function parameters at 18 years. High BMI trajectory was associated with high DNAm, which was further linked to decreased lung function at 18 years at all eight CpGs in males. High BMI trajectory was associated with increased lung function at 18 years, via high DNAm at 13 CpGs in females. At 6 (75%) of the 8 identified CpGs in males and 14 (87.5%) of the 16 identified CpGs in females, the same direction of indirect effects was observed at 26 years, but the indirect effects (in magnitude and/or statistical significance) at 26 years were weaker compared to 18 years, indicating that the mediating influence of DNAm at 10 years was likely to be reduced over time. Childhood BMI trajectories were associated with at least one lung function parameter in both sexes (Additional file 1: Table S1), which warranted the evaluation of mediating effects of DNAm. For females, the direct and indirect effects are opposite at most of the CpG sites, i.e., indirect effects being positive but direct effects being negative, or vice-versa (Table 2), which led to statistically insignificant total effects of BMI trajectories on lung function (i.e., no association between BMI trajectories and lung function). In males, the directions of direct and indirect effects are the same for FEV_1_ and for FVC, which are accumulated to a stronger total effect of BMI trajectory on lung function (Additional file 1: Table S1), while for FEV_1_/FVC, the direct and indirect effects are opposite and thus no total effects are observed. To our knowledge, this is the first study to examine the mediating effect of DNAm at pre-adolescence in the association of BMI trajectories in childhood with lung function at post-adolescence and young adulthood. The time ordered study design allowed investigation of the total effects of childhood BMI trajectory on post-adolescent and adulthood lung function parameters and whether and how DNAm played a role as mediator.

The genes mapped to identified mediating CpGs relevant to the pathophysiology of asthma and COPD. The gene expression of *LHFP* is correlated with *IL6R*, which plays an important role in asthma pathophysiology and obesity-related inflammation [60]. *DHX58* is differentially expressed between asthmatics and non-asthmatics [61] and it’s expression is increased in subjects with COPD [62], and in our study was associated with DNAm at cg00647820. *CAMTA1* has been demonstrated to be a *cis*-methQTL (methylation quantitative trait loci) using lung tissue samples of COPD cases and control subjects [63].

Additionally, assessment of biological relevance indicated a potential for epigenetic regulatory functionality on gene activities. The neighboring genes showing association of expression with DNAm at the identified CpGs included *TRAM1*, which has been shown to be correlated with FEV_1_ [64], and *DHRS3* whose expression is associated with smoke exposure, a strong risk factor for lung disease [65].

The Mendelian Randomization test further suggested that two of the 20 identified CpGs, cg19088553 in *GRIK2* and cg00612625 in *HPSE2*, were potential causal factors for FEV_1_ variation. The consistency of the mediation effects of these two CpGs between ages 18 and 26 years lends further support to the potential for causality. Although we were not able to assess the biological functionality of these CpGs due to the lack of expression data of their mapped genes (*GRIK2* and *HPSE2*) in whole blood, earlier studies have linked both genes with pulmonary diseases. *GRIK2* has been shown to have increased gene expression in cells isolated from individuals with COPD [66]. Differential methylation of *HPSE2* has been shown to be associated with FEV_1_ in older age [67] and genetic variants in *HPSE2* have been associated with childhood onset asthma [68]. Although promising findings, it is worth noting the limitations of the two-sample MR approach, including weak instrument biases towards the null and that confounders of risk factor-outcome association are not related to the genetic instrument [69]. Such limitations warrant caution in interpretation of the suggested potential causal effects inferred from our study.

The availability of lung function parameters at two key time points, post-adolescence and adulthood, allowed us to examine and compare mediating effects of pre-adolescence DNAm at both ages. In comparison to using BMI at discrete time points, BMI trajectories allow the visualization of the dynamics of BMI changes over time for certain groups of participants, with each group representing unique BMI patterns over time. The use of BMI trajectories allows for the simultaneous consideration of intensity, age of onset and duration of adiposity, which may improve the predictability of future BMI patterns. Further, we were able to examine if the identified mediating CpGs continue to mediate the association of childhood BMI trajectories with lung function in adulthood. The two BMI trajectories inferred in the IOWBC were based on goodness of fit criteria as well as the complexity of trajectory patterns. Since the birth cohort is population based, we expect that the trajectories reflect populations with similar features. On the other hand, demographics and age range in this study may be limiting factors in the validity of findings, and hence generalization of these trajectories and related findings to other populations should be implemented with caution. In one of our earlier studies by Ziyab et al. [70], four trajectories were identified, “normal”, “early persistent obesity”, “delayed overweight”, and “early transient overweight”, which had more detailed features compared to the two trajectories inferred in the current study. One of the potential reasons leading to such a difference is the time points used to infer trajectories. In this study, trajectories of BMI were constructed based on BMI at ages 1, 2, 4 and 10 years while Ziyab et al. evaluated trajectories using time points at 1, 4, 10 and 18 years. In addition, this study focused on subjects who did not have asthma at all the four ages (1, 2, 4, and 10 years), which not only led to a different study population but also different sample sizes compared to those in Ziyab et al. Considering our genome-scale analyses, in addition to a carefully planned screening process, to avoid power loss, we focused on parsimonious trajectories to detect epigenetic factors potentially associated with overweight or obesity in general. As we performed a carefully designed screening process, we deemed the CpGs included in the final model as pre-selected CpGs in terms of their impact on the association of BMI trajectory and lung function parameters. In such case, following the suggestion in the literature [71], multiple testing adjustment was not performed.

Some limitations are present in our study. We evaluated the contributions of each CpG site. These CpGs may be correlated and jointly impact lung function, which could not be addressed in this study. Also, DNA was extracted from peripheral blood cells while lung function is primarily affected by cells of the respiratory tract. Although DNAm in the blood cells is different to that of the respiratory system cells, concordance has been demonstrated between the two tissues [72]. Hence, CpGs relevant to lung function can be identified in the blood cells, which are more easily accessible. Although future studies are warranted to further examine the credibility of the identified CpGs, the consistency in the results at two ages, 18 and 26 years, indicate a role for epigenetics in the association of obesity and lung function.

## Conclusion

The identified CpGs have the potential to improve our understanding of the underlying biological pathways in the connection between obesity and lung function, with 2 CpGs (cg19088553 on gene *GRIK2* and cg00612625 on gene *HPSE2*) having the potential for causal association with lung function.

## Supplementary Information

**Additional file 1. Supplemental Tables**. Association of BMI trajectory and lung function via DNA methylation.

## Data Availability

The datasets analyzed during the current study are not publicly available but are available from the corresponding author on request.
